# Incremental clinical value of intraplaque neovascularization in predicting recurrent ischemic stroke

**DOI:** 10.1002/acn3.52255

**Published:** 2024-11-18

**Authors:** Liuping Cui, Ran Liu, Fubo Zhou, Bing Tian, Ying Chen, Yingqi Xing

**Affiliations:** ^1^ Department of Vascular Ultrasound, Xuanwu Hospital Capital Medical University Beijing China; ^2^ Department of Neurology The First Hospital of Jilin University Changchun China; ^3^ Beijing Diagnostic Center of Vascular Ultrasound Beijing China; ^4^ Center of Vascular Ultrasound Beijing Institute of Brain Disorders, Collaborative Innovation Center for Brain Disorders, Capital Medical University Beijing China

## Abstract

**Objective:**

Carotid intraplaque neovascularization (IPN) detected by contrast‐enhanced ultrasound (CEUS) is a risk factor for recurrent ischemic stroke. However, it is still unclear whether IPN can be used to accurately identify patients with recurrent ischemic stroke in clinical practice. Herein, we investigated the clinical predictive value of IPN for recurrent ischemic stroke in a real‐world setting.

**Methods:**

We enrolled 200 patients with ischemic stroke and atherosclerotic carotid stenosis who were followed up for 2 years. The endpoint was recurrent ischemic stroke. Cox regression and subgroup analyses were employed to assess whether treatment affected the relationship between IPN and recurrent ischemic stroke. The net classification index (NRI) and integrated discriminant improvement index (IDI) were used to validate the additional clinical value of IPN in identifying recurrent ischemic stroke.

**Results:**

During the 2‐year follow‐up, 36 patients experienced recurrent ischemic stroke. Cox regression analyses showed that IPN (grade 2), hypoechoic plaque, high homocysteine levels, and smoking were independent risk factors for recurrent ischemic stroke. Additional IPN evaluation may increase the NRI (0.512; 95% confidence interval [CI]: 0.083–0.624) and IDI (0.151; 95% CI: 0.010–0.213) for identifying high‐risk patients with recurrent ischemic stroke. In addition, in the subgroup undergoing revascularization, the proportion of IPN (grade 2) was significantly higher in patients with recurrent ischemic stroke than in patients with nonrecurrent ischemic stroke (*p* = 0.001).

**Interpretation:**

In clinical settings, IPN, assessed by CEUS, may provide additional clinical value for predicting recurrent ischemic stroke, helping to identify patients with ischemic stroke who require close follow‐up.

## Introduction

Atherosclerosis is a systemic inflammatory vascular disease that affects multiple vascular beds and increases the risk of stroke, cardiovascular events, or death. The Trial of Org 10172 in acute stroke treatment (TOAST) classification system classifies ischemic stroke into five types based on the etiology, of which 10–25% are atherosclerotic ischemic stroke.^1^, ^2^ Patients with atherosclerotic ischemic stroke have a higher risk of recurrent stroke than those with other stroke types.^3^ Therefore, strengthening residual risk control after an atherosclerotic ischemic stroke is crucial.

Carotid atherosclerosis is a typical manifestation of vascular diseases. It is also a surrogate marker of generalized atherosclerosis and a predictor of cardiovascular events.^4^ The latest guidelines suggest that atherosclerotic ischemic stroke is related to the degree of carotid stenosis and the composition and morphologic characteristics of carotid plaques.^5^ Recent studies have demonstrated a correlation between intraplaque neovascularization (IPN) and ischemic stroke recurrence.^6^, ^7^ However, it is not fully understood whether pharmacologic therapy affects the relationship between IPN and ischemic stroke recurrence in clinical settings. Additionally, a histological study based on a carotid endarterectomy cohort showed that plaque lipid composition, IPN, and intraplaque hemorrhage may increase the risk of future cardiovascular events.^8^ Studies also suggest that hypoechoic vulnerable plaques detected by carotid ultrasound are associated with restenosis and cardiovascular events after carotid revascularization.^9^, ^10^, ^11^ Currently, it is unclear whether contemporary medical management (pharmacologic therapy and revascularization) alters the predictive value of IPN for ischemic stroke recurrence in clinical practice.

To address this, in this study, we aimed to investigate whether IPN detected by contrast‐enhanced ultrasound (CEUS) provides additional clinical value for predicting recurrent ischemic stroke in patients with atherosclerotic ischemic stroke and whether IPN can accurately identify patients at high risk for recurrent ischemic stroke who require close follow‐up.

## Patients and Methods

### Study population

This was a dual‐center prospective observational study. Between January 2018 and August 2021, 200 patients with symptomatic carotid stenosis were consecutively enrolled from the First Hospital of Jilin University and Xuanwu Hospital of Capital Medical University. The inclusion criteria were as follows: (1) patients who had experienced an ischemic stroke and were confirmed to have a new ischemic lesion using computed tomography or magnetic resonance imaging; (2) patients diagnosed with atherosclerotic ischemic stroke following the TOAST classification. The exclusion criteria were as follows: (1) poor‐quality carotid ultrasound images that could not be analyzed; (2) the presence of isolated posterior circulation infarcts; (3) severe infections, tumors, hepatorenal failure, or respiratory failure; and (4) loss to follow‐up.

The study protocol conformed to the ethical guidelines of the 1975 Declaration of Helsinki. This study was approved by the Ethics Committees of two centers. Written informed consent was obtained from all patients.

### Carotid ultrasonography and CEUS

Experienced sonographers (CY and XYQ) performed all operations following the Chinese stroke vascular ultrasound examination guidelines. The common carotid artery, bifurcation, and internal carotid artery were scanned in the transverse and longitudinal views with the patient in the prone position. The Aplio500 (Toshiba, Japan) and Epiq7 (Philips, Netherlands) ultrasound machines were used in this study.

The degree of stenosis was categorized as <50%, 50–69%, and 70–99%, following the Radiological Society of North America classification.^12^ The plaques were categorized into the following five types according to the Gray–Weale scale: type I, homogeneous hypoechoic; type II, predominantly hypoechoic; type III, isoechoic; type IV, predominantly hyperechoic; and type V, severely calcified with acoustic shadowing.^13^ When a plaque was observed, its location, size, and echogenicity were recorded. If multiple plaques were present, the thickest was used as the target plaque, and CEUS was performed.

**Figure 1:**
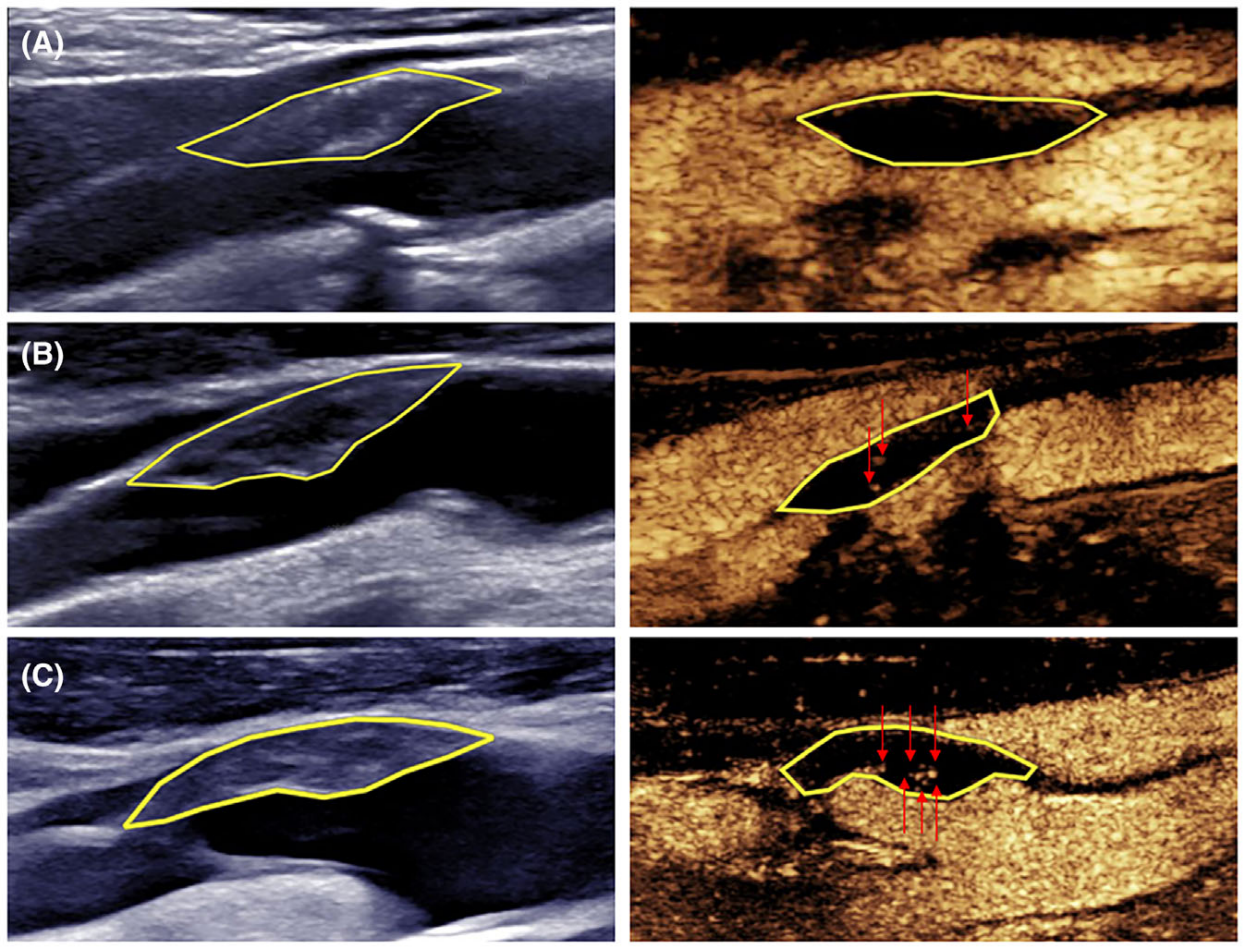
IPN grading: (A) Grade 0: No enhanced microbubbles within the plaque; (B) Grade 1: A small number of enhanced microbubbles within the plaque; (C) Grade 2: A large number of enhanced microbubbles within the plaque, penetrating its core. Red arrows and yellow lines indicate enhanced microbubbles and plaques, respectively.

The CEUS examination followed a previously described procedure,^14^ and all the images were saved on a hard disk for offline analysis. Plaque vulnerability was assessed using a semiquantitative approach. IPN and plaque ulcers were identified based on the direction of contrast diffusion and microbubble enhancement characteristics. IPN detected by CEUS was characterized by contrast diffusion from the artery adventitia toward the lumen, with enhanced microbubbles appearing as dots or short lines. In contrast, ulcers detected by CEUS were characterized by mass contrast flow from the arterial lumen into the concave side of the plaque‐lumen interface, accompanied by carotid lumen enhancement.^15^ IPN was categorized into the following three grades: grade 0, no contrast‐enhanced signal observed within the plaque; grade 1, a small amount of contrast‐enhanced signal observed in the shoulder and/or adventitial side; and grade 2, substantial contrast‐enhanced signal observed within the plaque (Fig. 1). Two sonographers (CY and XYQ) analyzed the images, and in cases of disagreement, consensus was reached via discussion.

### Clinical characteristics and follow‐up

The following clinical data were recorded: (1) age and sex; (2) risk factors, including hypertension, diabetes, smoking, drinking, and family history; (3) serological indicators, including cholesterol, triglyceride, high‐density lipoprotein, low‐density lipoprotein, fasting blood glucose, homocysteine, and high‐sensitivity C‐reactive protein levels; (4) whether the patients underwent carotid revascularization; and (5) medication adherence. Good adherence was defined as patients taking their medications for at least 80% of the days during the study period.^16^

A physician who remained blinded to patients' baseline information followed up with patients every 6 months until the study ended. The endpoint event was ischemic stroke recurrence.

### Statistical analysis

Statistical analyses were performed using IBM SPSS Statistics (version 26.0; IBM Corp., Armonk, NY, USA) and R version 4.2.2 (R Foundation for Statistical Computing, Vienna, Austria). Statistical significance was set at *p* < 0.05. Normally distributed continuous variables are expressed as the mean ± standard deviation and non‐normally distributed continuous variables as the median (interquartile range). Analyses were performed using the Student's *t*‐test or nonparametric rank sum test, respectively. Categorical variables are expressed as frequencies (percentages) and were analyzed using the chi‐squared test. The factors with statistical differences between the two groups (*p* < 0.05) were included in the multivariate Cox proportional hazards regression model to analyze the association between IPN and recurrent ischemic stroke, and a receiver operating characteristic curve was constructed to identify patients at high risk for recurrent ischemic stroke. The forest plot was used to display independent predictors of recurrent ischemic stroke. The Delong test was used to compare the area under the curves (AUCs). Finally, the net reclassification index (NRI) and the integrated discriminant improvement index (IDI) were employed to demonstrate the incremental value of IPN in assessing recurrent ischemic stroke in patients.

In addition, subgroup analyses were performed to assess whether potential covariates confounded the relationship between IPN and recurrent ischemic stroke.

## Results

### Patients and outcomes

**Figure 2:**
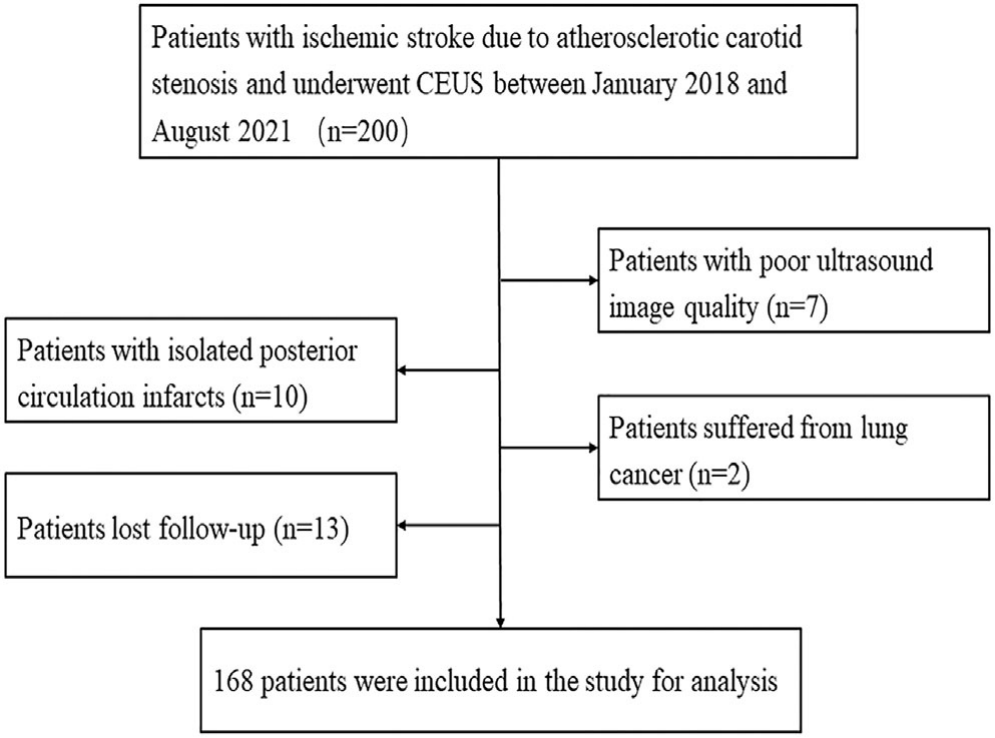
The study flowchart.

In total, 200 patients with symptomatic carotid artery stenosis were recruited. However, 32 patients were excluded for the following reasons: Seven had poor ultrasound image quality, 10 were diagnosed with isolated posterior circulation infarcts, two had lung cancer, and 13 were lost to follow‐up (Fig. 2). Therefore, 168 patients (145 men and 23 women, with a mean age of 64.2 ± 8.0 years) who met the inclusion criteria were included in the study. During the 2‐year follow‐up period, 36 patients experienced recurrent ischemic stroke.

### Patient baseline characteristics

The patients were divided into the “recurrent” (*n* = 36) and “non‐recurrent” groups (*n* = 132) based on whether they had recurrent ischemic stroke. Table 1 presents the baseline patient characteristics. The two groups showed no statistically significant differences in the demographic characteristics. The recurrent group had a significantly higher proportion of patients who smoked than the non‐recurrent group (*p* = 0.001). However, other vascular risk factors were similar in both groups. Furthermore, patients in the recurrent group had significantly higher cholesterol (*p* = 0.023), low‐density lipoprotein (*p* = 0.014), fasting blood glucose (*p* = 0.015), homocysteine (*p* = 0.001), and high‐sensitivity C‐reactive protein (*p* = 0.011) levels than patients in the non‐recurrent group.

**Table 1:** Baseline characteristics of patients (*N* = 168).

	Non‐recurrent (*n* = 132)	Recurrent (*n* = 36)	*p*‐value
Demographics			
Age, year, mean ± SD	63.7 ± 7.4	66.4 ± 9.4	0.069
Sex, man, *n* (%)	114 (86.4)	31 (86.1)	0.969
Risk factors, *n* (%)			
Hypertension	88 (66.7)	25 (69.4)	0.753
Diabetes	39 (29.5)	14 (38.9)	0.285
Smoking	68 (51.5)	30 (83.3)	**0.001**
Drinking	56 (42.4)	15 (41.7)	0.935
Family history	57 (43.2)	13 (36.1)	0.446
Serological indicators			
Cholesterol, mean ± SD	3.6 ± 0.8	4.0 ± 1.0	**0.023**
Triglycerides, mean ± SD	1.4 ± 0.7	1.6 ± 0.6	0.052
HDL, median (IQR)	1.0 (0.3)	1.0 (0.4)	0.058
LDL, mean ± SD	2.2 ± 0.7	2.5 ± 0.7	**0.014**
FBG, median (IQR)	5.3 (1.4)	6.1 (2.4)	**0.015**
Homocysteine, mean ± SD	13.2 ± 4.3	15.9 ± 4.4	**0.001**
hs‐CRP, median (IQR)	3.0 (2.7)	3.2 (0.9)	**0.011**
Treatment, *n* (%)			
Antiplatelet drugs	119 (90.2)	23 (63.9)	0.081
Lipid‐lowering drugs	43 (32.6)	14 (38.9)	0.060
Antihypertensive drugs	80 (60.6)	17 (47.2)	**0.010**
Hypoglycemic drugs	36 (27.3)	10 (27.8)	0.129
Carotid revascularization	62 (47.0)	10 (27.8)	**0.039**
Plaque characteristics			
Degree of stenosis, *n* (%)			0.451
50–69%	46 (34.8)	15 (41.7)	
70–99%	86 (65.2)	21 (58.3)	
Plaque echogenicity, *n* (%)			**0.001**
Types I and II	55 (41.7)	27 (75.0)	
Types III and IV	77 (58.3)	9 (25.0)	
IPN, *n* (%)			**<0.0001**
Grades 0 and 1	78 (59.1)	9 (25.0)	
Grade 2	54 (40.9)	27 (75.0)	

Values in bold indicate *p* < 0.05.

FBG, fasting blood glucose; HDL, high‐density lipoprotein; hs‐CRP: high‐sensitivity C‐reactive protein; IPN, intraplaque neovascularization; IQR, interquartile range; LDL, low‐density lipoprotein; SD, standard deviation.

Significantly more patients in the non‐recurrent group than those in the recurrent group received carotid revascularization (*p* = 0.039). For patients with vascular risk factors, no significant difference was noted between the recurrent group and the non‐recurrent group in the use of antiplatelet drugs, lipid‐lowering drugs, or hypoglycemic drugs. The proportion of patients taking antihypertensive drugs was significantly higher in the recurrent group than in the non‐recurrent group (*p* = 0.010).

### Plaque characteristics

Regarding plaque characteristics, the recurrent group exhibited a significantly higher number of culprit plaques with hypoechoicity (*p* = 0.001) and grade 2 IPN (*p* < 0.0001) than the non‐recurrent group. The two groups showed no significant difference in the degree of stenosis. For the IPN evaluation, there was good consistency between the inter‐ and intra‐observer agreements (kappa values of 0.786 and 0.869, respectively).

### Analyses of risk associations and risk prediction

**Figure 3:**
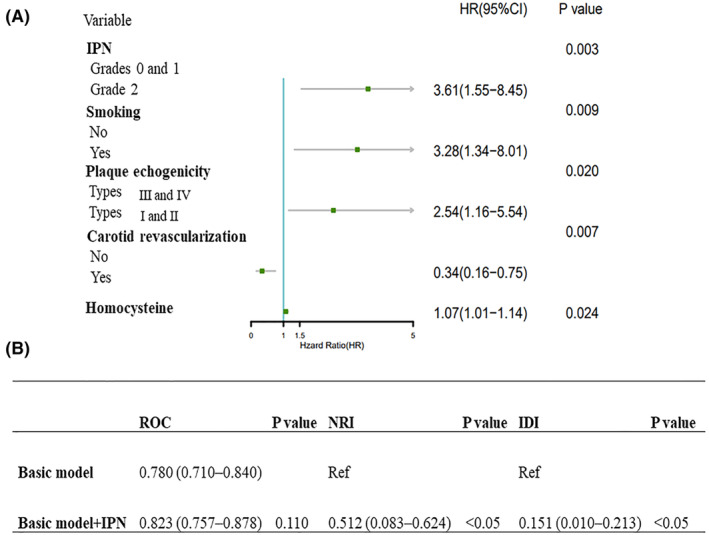
The multivariate Cox proportional hazards regression. (A) The forest plot shows the independent predictors for patients with recurrent ischemic stroke. (B) Comparison of accuracy between the basic model and the basic model combined with IPN in identifying patients at risk for recurrent ischemic stroke. IPN, intraplaque neovascularization.

Figure 3 presents the results of the multivariate Cox proportional hazards regression model. Grade 2 IPN (hazard ratio [HR]: 3.61; 95% confidence interval [CI]: 1.55–8.45; *p* = 0.003), smoking (HR: 3.28; 95% CI: 1.34–8.01; *p* = 0.009), hypoechoic plaques (HR: 2.54; 95% CI: 1.16–5.54; *p* = 0.020), and high homocysteine levels (HR: 1.07; 95% CI: 1.01–1.14; *p* = 0.024) were strongly associated with recurrent ischemic stroke. Carotid revascularization (HR: 0.34; 95% CI: 0.16–0.75; *p* = 0.007) reduced the risk of recurrent ischemic stroke.

Furthermore, we analyzed the clinical value of IPN in identifying patients at high risk of recurrent ischemic stroke. The results indicated that the AUC of the combination of smoking, hypoechoic plaques, and high homocysteine levels (basic model) was 0.780 (95% CI: 0.710–0.840), while the AUC of the basic model and IPN combination was 0.823 (95% CI: 0.757–0.878), with no statistically significant difference (*p* = 0.110).

The NRI of the basic model and IPN combination was 0.512 (95% CI: 0.083–0.624), with an IDI of 0.151 (95% CI: 0.010–0.213). The two indicators demonstrate significant differences, indicating that the basic model and IPN combination exhibits a greater capacity for discrimination and risk reclassification than the basic model alone.

### Subgroup analysis

**Figure 4:**
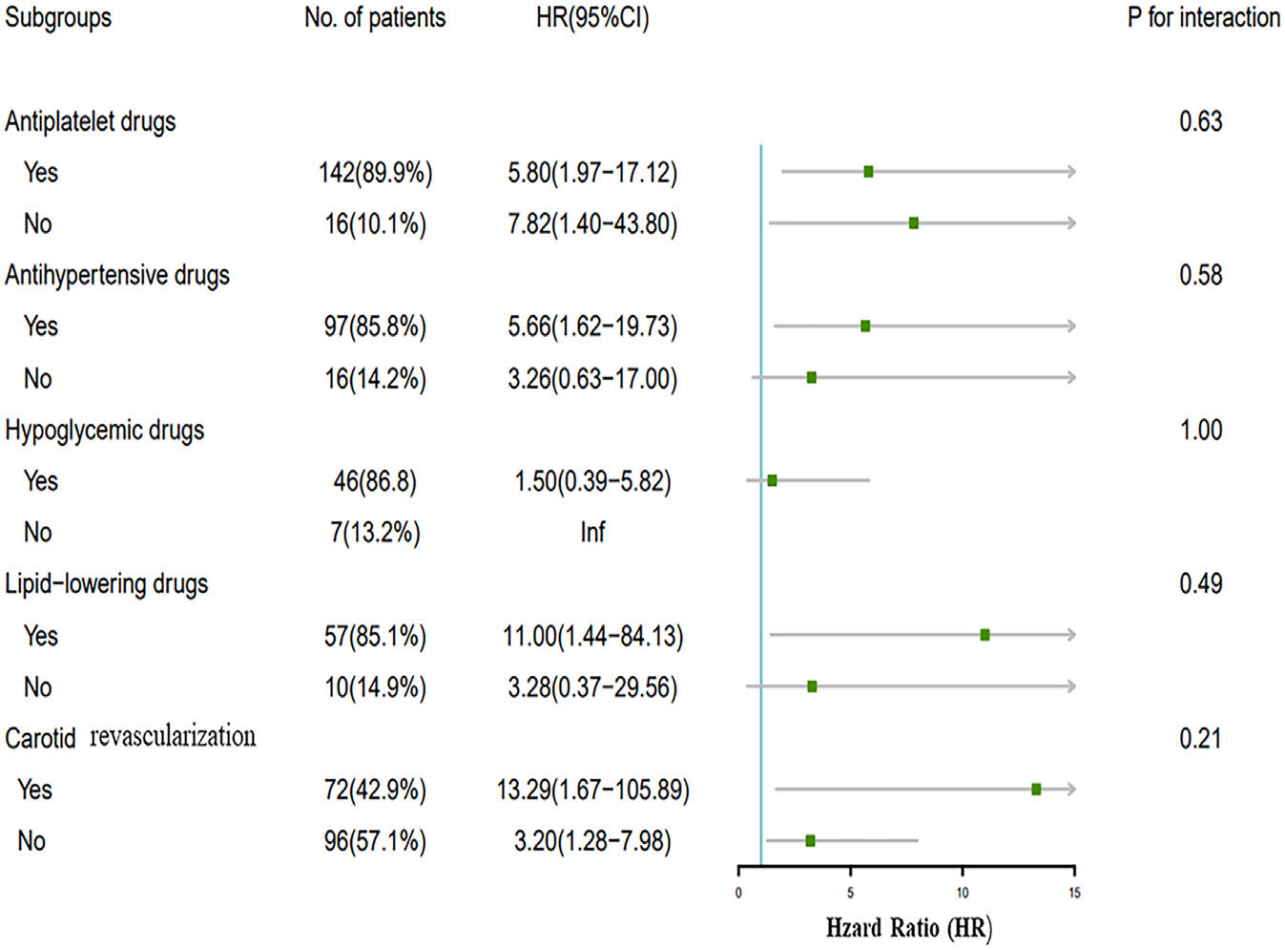
The subgroup analysis between IPN and endpoint event. IPN, intraplaque neovascularization.

The subgroup analysis revealed the influence of treatment on the relationship between IPN and recurrent ischemic stroke. No significant interaction was observed between IPN and these potential factors (various treatments) for recurrent ischemic stroke when stratified by antiplatelet drugs, lipid‐lowering drugs, antihypertensive drugs, hypoglycemic drugs, and carotid revascularization (p > 0.05) (Fig. 4).

### Additional analysis – carotid revascularization and IPN

**Figure 5:**
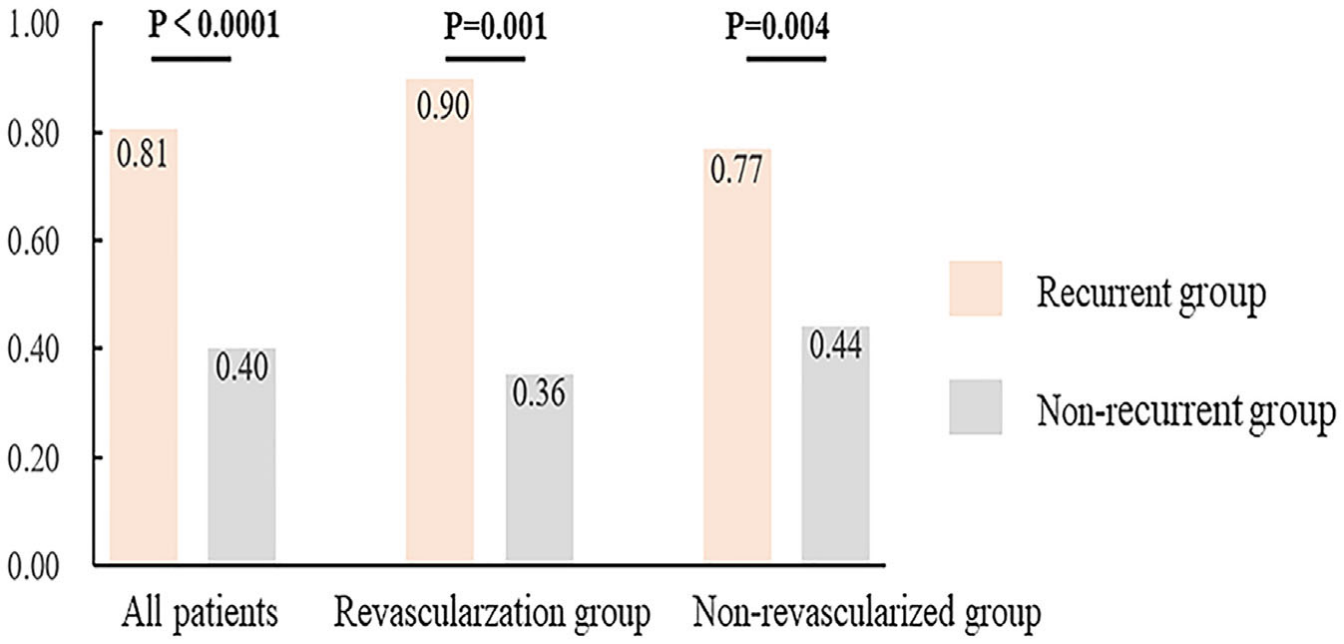
Comparison of IPN between patients with recurrent and non‐recurrent ischemic stroke in different subgroups. IPN, intraplaque neovascularization.

Figure 5 illustrates the association between IPN and recurrent ischemic stroke in different subgroups. In the nonrevascularized group, the proportion of patients with IPN (grade 2) was higher in patients with recurrent ischemic stroke than in patients with non‐recurrent ischemic stroke (77% vs. 44%, *p* = 0.001). Similarly, in the revascularization group, a significantly higher proportion of patients with recurrent ischemic stroke than patients with non‐recurrent ischemic stroke had IPN (grade 2) (90% vs. 36%, *p* = 0.001).

## Discussion

In this study, we demonstrate that the IPN detected by CEUS may provide additional clinical value in predicting recurrent ischemic stroke, assisting clinicians in identifying and closely following up patients with high‐risk for recurrent ischemic stroke in clinical practice.

The relationship between IPN and adverse outcomes in patients with ischemic stroke has been extensively studied. However, the complexity of the clinical practice setting surpasses that of the research environment. Hence, we investigated the clinical applicability of IPN for the risk stratification of patients with ischemic stroke in a real‐world clinical setting. In this study, which controlled for vascular risk‐reducing medications, such as antihypertensive, hypoglycemic, lipid‐lowering, and antiplatelet drugs, multivariate Cox proportional hazards regression modeling analyses showed that IPN remained an independent predictor of ischemic stroke recurrence, even when the patients received medical management. A previous study demonstrated a rapid and significant reduction in IPN after 3 months of treatment with rosuvastatin.^17^ However, the study was not placebo‐controlled and had a small sample size, Prompting the need for larger randomized controlled trials to confirm the results. Furthermore, in our study, carotid revascularization was not associated with IPN and was entered into the final model as a protective factor. In addition, we conducted a subgroup analysis to examine the relationship between IPN and ischemic stroke recurrence. The results demonstrated that, regardless of whether carotid artery revascularization was performed, IPN was an independent risk factor for ischemic stroke recurrence. Several findings support our hypothesis that patients with one vulnerable plaque are at higher risk of developing vulnerable plaques at other vascular sites.^8^, ^18^ Hellings et al. demonstrated that increased IPN density is independently associated with an increased risk of future systemic cardiovascular events, including carotid, coronary, and peripheral ischemia, even after plaque removal.^19^

The combined application of multiple parameters is expected to improve the validity of predicting the occurrence of ischemic events and reduce the residual risk in patients with ischemic stroke. Carotid ultrasonography has become the primary screening method for atherosclerotic diseases owing to its convenience, speed, and affordability. He et al. used vascular ultrasonography to determine if vulnerable plaque features, such as IPN and plaque stiffness, were associated with an increased risk of poor short‐term functional outcomes in patients with ischemic stroke due to recent carotid atherosclerosis.^20^ They found that combining multiple vulnerability features had higher accuracy in predicting poor short‐term functional outcomes after stroke. In addition, Wu et al. combined B‐mode ultrasonography and CEUS to analyze the characteristics of vulnerable carotid plaques and established a carotid plaque score for the risk stratification of patients with ischemic stroke.^21^ Our study confirms and extends previous research suggesting that, compared with the basic model (hypoechoic plaques, smoking, and high homocysteine levels) alone, the combination of IPN and the basic model may more accurately identify patients at high risk for ischemic stroke recurrence, regardless of treatment.

We identified smoking and high homocysteine levels as risk factors in the final model. Smoking and high homocysteine levels are risk factors for recurrent ischemic stroke.^22^, ^23^ This association may be due to underlying mechanisms such as inflammation, atherosclerosis, and increased platelet aggregation, all of which accelerate stroke development.^24^ Inflammation has been reported to affect carotid plaque and IPN formation. Lack of oxygen and increased metabolic demand within the plaque cause neovascularization to proliferate. Neovascular permeability is critical in inflammatory infiltration promotion and inflammation level elevation, which further increases vascular permeability, creating a positive feedback loop.^25^, ^26^ These studies emphasized atherosclerosis as a chronic systemic inflammatory response.^27^

This study has some limitations that warrant consideration. First, the number of participants with outcome events was small. Future multicenter, large‐sample studies are required to validate these findings. Second, many male patients were included in this study, which may be a bias as there is a higher incidence of ischemic events in men. Third, there was a high degree of operator variability in the carotid ultrasonography. However, the ultrasonographers standardized the examination following the same protocols, ensuring good inter‐operator consistency. Finally, only the thickest plaques were considered, which could introduce potential spectral bias.

In conclusion, IPN assessed by CEUS may provide additional value in the risk stratification of high‐risk patients with ischemic stroke recurrence among patients with atherosclerotic ischemic stroke. IPN may also facilitate the close follow‐up of high‐risk patients.

## Funding Information

This study was supported by the National Key Research and Development Projects (2022YFC3602400) and Beijing Hospitals Authority Youth Programme (QML20230814) and the National Natural Science Foundation of China (Grant No. 82102066).

## Conflict of Interest

The authors declare that they have no conflicts of interest.

## Author Contributions

**Conceptualization:** Liuping Cui, Ying Chen, and Yingqi Xing; **Data curation:** Ying Chen and Yingqi Xing; **Methodology:** Ran Liu, Fubo Zhou, and Bing Tian; **Formal analysis and investigation:** Ran Liu and Fubo Zhou; **Writing – original draft preparation:** Liuping Cui and Ying Chen; **Writing – review, and editing:** Ying Chen and Yingqi Xing; **Funding acquisition:** Fubo Zhou, Ying Chen, and Yingqi Xing; **Supervision:** Ying Chen and Yingqi Xing.

## Data Availability

Data are available on reasonable request. All supporting data within the article are available on reasonable request from a qualified investigator.
